# Supplementary material for: ProteinWeaver: A webtool to visualize ontology-annotated protein networks
Source: PLoS One. 2025 Sep 5;20(9):e0331280. doi: 10.1371/journal.pone.0331280 (PMC12413088; doi:10.1371/journal.pone.0331280)
Supplement: Attachment — Submitted filename: ProteinWeaver Response to Reviewers.pdf [file pone.0331280.s002.pdf]

Dear *PLOS ONE* Editor,

Thank you for your comments and the two reviews. Our point-by-point responses to your requests and the reviewers' comments are indicated in **blue.**

Thank you,
Anna Ritz, on behalf of all co-authors

Requests by the Editor:

1. Please ensure that your manuscript meets PLOS ONE's style requirements, including those for file naming.
We have followed the PLOS ONE style requirements.

2. Please expand the acronym "DBI" (as indicated in your financial disclosure) so that it states the name of your funders in full. This information should be included in your cover letter; we will change the online submission form on your behalf.
The funding source is the National Science Foundation - Division of Biological Infrastructure (referenced by the acronym DBI in the initial submission).

3. We note that you have provided funding information that is not currently declared in your Funding Statement. Please remove any funding-related text from the manuscript and let us know how you would like to update your Funding Statement.
We removed the funding information from the acknowledgements.

Currently, your Funding Statement reads as follows:
This work was supported by a National Science Foundation (https://www.nsf.gov/) grant NSF-DBI-1750981 awarded to AR. The funders not play any role in the study design, data collection and analysis, decision to publish, or preparation of the manuscript.
This is correct - thanks.

4. At this time, please upload the minimal data set necessary to replicate your study's findings to a stable, public repository (such as figshare or Dryad) and provide us with the relevant URLs, DOIs, or accession numbers that may be used to access these data.
All datasets have been deposited to Zenodo (https://zenodo.org/records/15843724). The Zenodo tag is also added to our GitHub repository, which contains the source code for the website.

5. Please include captions for your Supporting Information files at the end of your manuscript, and update any in-text citations to match accordingly. Please see our Supporting Information guidelines for more information: http://journals.plos.org/plosone/s/supporting-information.

We have added a caption for our Supplementary Information at the end of the manuscript, and updated all in-text citations according to the instructions for authors.

6. Please review your reference list to ensure that it is complete and correct. Any changes to the reference list should be mentioned in the rebuttal letter that accompanies your revised manuscript. If you need to cite a retracted article, indicate the article's retracted status in the References list and also include a citation and full reference for the retraction notice.

We added a number of new citations in response to reviewers:

I. A paper that describes mixed motifs was recently published - we have added this citation to the manuscript:

   [26] Altaf Barelvi, Oliver Anderson, Anna Ritz, GRPhIN: Graphlet Characterization of Regulatory and Physical Interaction Networks, Bioinformatics Advances, 2025;, vbaf176.

II. We updated one of the case studies in the Results section, which removes 1 citation and adds 3 citations about the connection between microtubule bundle formation and embryogenesis. The new citations are listed below:

   [38] Liu R, Abreu-Blanco MT, Barry KC et al. Wash functions downstream of Rho and links linear and branched actin nucleation factors. Development 2009;136:2849–60.

   [39] Wilkin MB, Whiteford R, Akbar T et al. The First Defined Null Allele of the Notch Regulator, a Suppressor of Deltex: Uncovering Its Novel Roles in Drosophila melanogaster Oogenesis. Biomolecules 2024;14:522.

   [40] Li J, Li W, Calhoun HC et al. Patterns and functions of STAT activation during Drosophila embryogenesis. Mech Dev 2003;120:1455–68.

   Removed citation: Abrieu A, Liakopoulos D. How Does SUMO Participate in Spindle Organization? Cells 2019;8:801.

III. We added two new species, which added 2 citations to the Supplement:

   [2] Jin J, He K, Tang X et al. An Arabidopsis Transcriptional Regulatory Map Reveals Distinct Functional and Evolutionary Features of Novel Transcription Factors. Molecular Biology and Evolution 2015;32:1767–73.

   [17] Salgado H, Gama-Castro S, Lara P et al. RegulonDB v12.0: a comprehensive resource of transcriptional regulation in E. coli K-12. Nucleic Acids Res 2024;52:D255–64.

Comments from Reviewer #1:

1. Please clarify the reasons of choosing Yen's and breadth-first search algorithms.

We have adjusted the paragraph in the Queried Subnetworks section of the Methods to read as follows, with bolded sentences referring to your comment:

"The fundamental feature of ProteinWeaver is the ability for a user to enter a query protein *s* and a GO term *t* and visualize the connections from *s* to proteins annotated to t. **We use the notion of *paths* in the network to visualize nearby connections to a query protein. Paths from *s* ensure that the subnetwork is connected to the query protein and allows a tunable parameter for the user to view increasingly larger subnetworks.** When we visualize the subnetwork, we do not include the GO term t, ending the path at the proteins annotated to t (e.g., the square blue nodes in Fig 2B). Users specify the size of the subnetwork returned by selecting a small integer *k* (which typically ranges between 5 and 25). ProteinWeaver has two different modes for visualizing subnetworks with parameter *k*. **In the first mode, "K Unique Paths," ProteinWeaver returns the *k* shortest paths from *s* to nodes annotated to *t* (in terms of the number of edges). K Unique Paths will first visualize immediate neighbors of *s* that are annotated to *t*, then will visualize nodes further from *s* as the user increases the parameter *k*. Yen's *k*-shortest paths algorithm efficiently computes the *k* shortest loopless paths from *s* to nodes annotated to *t* [36]. In the second mode, "K Unique Nodes,"' ProteinWeaver visualizes the *k* nodes annotated to *t* that are the closest to *s* (in terms of shortest path length). A simple breadth-first search from *s* to reachable nodes annotated to *t* is sufficient for this mode.** For both modes, increasing the value of *k* increases the size of the visualized network. More information about the algorithms used for subnetwork generation can be found in Section A1.5 in File S1."

2. It is better to discuss the used algorithms and their potentials more in the results and discussion.

Based on this comment, we moved the two case studies earlier in the Results to showcase ProteinWeaver's queries and illustrate the difference between the two modes of visualization (K Unique Paths vs. K Unique Nodes). Both case studies now include views of both modes. Additionally, we added the following sentences to compare the two views.

"...The two modes, "K Unique Paths" and "K Unique Nodes,"' visualize subnetworks with different network topologies: both modes use $k=15$, but 15 paths reach 10 nodes annotated to microtubule bundle formation (Figure 5A). On the other hand, a network that reaches 15 GO term-associated nodes results in a larger network with seven intermediary proteins (Figure 5B)..."

"...In this example, Gdf6a is a member of D/V pattern formation (denoted by the red square in Figure 6); ProteinWeaver can be used to find additional connections from Gdf6a to other members of the same GO term. Again, the mode of visualizing k=15 unique paths reaches

fewer nodes annotated D/V patterning than the mode of visualizing the connections needed to reach k=15 nodes annotated to D/V patterning…."

3. In this 5-model organisms based tool, E. coli is totally neglected. Although the authors brought the topic in Discussion section and trying to justify it, the reasons of this negligence were not enough.

We have added two new species, including *E. coli* and *A. thaliana,* to bring the list to seven included species. We have updated tables and figures throughout the manuscript and we have updated the data sources in the supplement. This is a major addition to the manuscript, and we hope these new species will make ProteinWeaver more attractive to the broader scientific community.

Comments from Reviewer #2:

1. It is highly recommended to make a logical relationship between the following paragraph of "Introduction" section and the previous one, and highlight the importance of your study.
"We present ProteinWeaver, a molecular interaction network visualization tool that generates subnetworks of physical and regulatory interactions based on a protein and a biological function of interest for non-human model organisms. In contrast to previous tools, ProteinWeaver links proteins to relevant biological processes, provides customizable network visualizations, and encourages interactive network exploration. To our knowledge,"
We have modified the first sentence of this paragraph to read "To address these challenges with existing tools, we developed ProteinWeaver,..."

2. With reference to this sentence of "Introduction" section: "Currently, ProteinWeaver supports a prokaryote (the Gram-positive bacterium Bacillus subtilis subsp. subtilis str. 168), a single-celled eukaryote (the brewer's yeast Saccharomyces cerevisiae S288C), two morphologically-distinct invertebrates (the fruit fly Drosophila melanogaster and the nematode Caenorhabditis elegans), and a vertebrate (the zebrafish Danio rerio)…." : It is very informative to select a representative from various organisms. However, it is highly encouraged to include a representative of viruses such as SARS-CoV-2 to make a comprehensive server that allow this valuable analysis workflow to users.
Virus-host interactions are a very interesting and important area of study; however, ProteinWeaver currently focuses on physical and regulatory interactions from a *single* organism at a time. A future version might combine SARS-CoV-2 and human interactomes, but it is out of scope for this paper.

3. Some errors exist in the manuscript such as "single-celled" which should be replaced by "unicellular". Moreover, all scientific names should be written in italics.
"Single-celled" and "unicellular" are synonyms, so we did not make this change. All scientific names are now written in italics.

4. The Saccharomyces cerevisiae is considered as the unicellular eukaryote, but the yeasts are mainly considered as a prokaryote.
S. cerevisiae is a eukaryote, and all yeasts are eukaryotes. We are unsure what this comment refers to.

5. Table captions should be inserted above the Tables. Moreover, please cite this Table below the related text. It is not common to start the manuscript section with a table or figure.
We have moved the caption to be above the table and it now appears within a section.

6. Some grammar issues and vague sentences exist. So, it is highly recommended to revise the manuscript accordingly and resolve this issue.
We have reorganized and revised the manuscript for clarity.

7. Some repetitive structures exist throughout the manuscript such as the following one in “Materials and Methods” section: "Currently, ProteinWeaver supports a prokaryote (the Gram-positive bacterium Bacillus subtilis subsp. subtilis str. 168), etc...." The authors are highly encouraged to place this information here and remove it from introduction.
We have removed some repetitive information. We have also moved some of the Results in the previous submission to the Methods section.

8. In “2.2.” section, Figure 3.A should be written as Figure 2.
We have updated the figure order and have also ensured that they are properly referenced in the main text.

9. It is not common to state both full structure and abbreviation of each term after the first definition. Please revise the manuscript.
We believe this is helpful for the readers, so we have left it in.

10. It is not common to discuss the literatures in results section, unless results and discussion being mixed.
Our results section highlights two case studies so we have kept this structure.

11. It is highly recommended to revise lengthy sentences like these: “We assessed the four methods by generating a dataset of 1,000 positives, which were selected by randomly choosing a protein-GO term edge and modifying G to remove that specific edge. We expect these nodes to have high confidence in their membership with the GO term. For every positive (protein-GO Term pair), we selected 100 negative proteins by identifying proteins that are (1) near the positive protein when considering PPI and regulatory edges only, (2) are not connected to the positive GO term, and (2) have approximately the same degree as the positive node.”. Such structures could confuse readers. Please revise the manuscript and resolve this issue accordingly.
The quoted text includes three sentences, we assume the reviewer is talking about the last one quoted. Some of these sentences need to explain some technical details for reproducibility, and

we have tried to make them as clear as possible. We have kept this sentence as-is with some minor modifications.

12. It might that large explanations like this, with the nested examples confuse the readers and decrease the value of the server usage. It is highly recommended to revise the manuscript and resolve the similar structures like this: "We found that RandomWalk had a nearly perfect ROC AUC across all species (Figure 6. RandomWalk also had the highest precision at varying recall values and it drops off precision at higher recall values. The rest of the methods performed considerably worse than RandomWalk in the ROC AUC analysis. Between the methods, their ranks varied in different species analysis for both the inferred and non-inferred networks. For example, the degree method was ranked second in the ROC value for C. elegans, however was ranked 4th for the yeast dataset. The Precision/Recall values for the One-Hop GO Overlap and Degree methods noticed an increase when using the inferred networks. The significance of this increase varied among the methods and which species. For example, the degree method noticed the largest Precision/Recall increase in the C. elegans dataset. The Hypergeometric method, however, did not show any noticeable difference between the Precision/Recall values across all the species when using the inferred and non-inferred networks."
We have re-written this paragraph in the Results section. It now reads:

"For each species, we plotted Receiver Operator Characteristic (ROC) and precision-recall curves (Fig 7 and Fig 8). The RandomWalk method (in orange) dominates in both metrics across all species: RandomWalk has a nearly perfect ROC AUC across all species and also has the highest precision at larger recall values. The rest of the methods performed considerably worse than RandomWalk. After RandomWalk's ROC AUC of 0.99, the next best ROC AUC is 0.77 (e.g., the Hypergeometric method in *S. cerevisiae* and the One-Hop GO Overlap in E. coli, Fig 7). The story is similar for PR AUC: RandomWalk's worst PR AUC is 0.77, whereas the next best PR AUC is 0.39 (e.g, the Degree method in *D. rerio*). The Degree method seemed to be better than the other two for *C. elegans* and *D. rerio*, suggesting degree bias in these species. Additionally, the One-Hop GO Overlap method seemed to be better than the other two for *S. cerevisiae*. However, the performance of all three methods are much poorer compared to the RandomWalk approach."

13. The manuscript has many vague structures like "This could be because adding inferred protein-GO edges did not affect the overall hypergeometric equation used to calculate their scores. We also ran these methods on the graphs with only directly-annotated networks; while the relative ordering of the three comparator methods changed, the random walk approach remained superior". Please revise whole document and correct it.
We have significantly revised and reorganized the manuscript. The stated text now reads:

"The background network includes directly annotated protein-GO term pairs as well as inferred annotations between proteins and more general GO terms (see Methods). One reason the RandomWalk might work so well is that it could rely on the inferred protein-GO term pairs rather than the direct annotations. To check this, we also ran the four protein function prediction

methods on the graphs with only directly-annotated networks; while the relative ordering of the three comparator methods changed, the random walk approach remained superior (Section A3.3 in File S1). Furthermore, we tested the performance of RandomWalk on background networks that only consisted of protein-GO term annotations (removing the protein interactions) and found that RandomWalk performed only slightly worse in terms of ROC and precision-recall (Section A3.4 in File S1).”

14. It is better to mention case studies in materials and methods section and only state the results here.
We opted to keep the case studies together in the Results section, rather than have it split across the Methods and Results sections.

15. It is not necessary to redundant explanations such as the microtubule or BMPs importance.
Given PLOS ONE’s broad audience, we think that brief explanations of proteins are in order. We have kept them in.

16. All phrases about the gathered results should be write in past tense.
The sentence about data collection is written in past tense: “For each organism, we collected experimental, text-mined, and database-validated protein and genetic interactions, as well as Gene Ontology (GO) annotations (Section A1.1 in S1 File).” Algorithms tend to be written in present tense, so we have kept that.

17. The authors have been provided a valuable web-server. However, it is recommended to improve the graphical representation.
We are unclear about what this comment refers to. We have had many users give feedback on the interface, which has gone through multiple organizational changes. We will continue to improve on the interface as we develop ProteinWeaver, but we believe that it is sufficient for the current manuscript.
